# Metabolic changes may precede proteostatic dysfunction in a *Drosophila* model of amyloid beta peptide toxicity

**DOI:** 10.1016/j.neurobiolaging.2016.01.009

**Published:** 2016-05

**Authors:** Stanislav Ott, Anastasia Vishnivetskaya, Anders Malmendal, Damian C. Crowther

**Affiliations:** a Department of Genetics, University of Cambridge, Cambridge, UK; b Faculty of Health and Medical Sciences, Department of Biomedical Sciences, University of Copenhagen, Copenhagen N, Denmark; c Neuroscience IMED, MedImmune Limited, Granta Park, Cambridge, UK

**Keywords:** *Drosophila*, Aging, Metabolomics, Amyloid beta, Proteostasis

## Abstract

Amyloid beta (Aβ) peptide aggregation is linked to the initiation of Alzheimer's disease; accordingly, aggregation-prone isoforms of Aβ, expressed in the brain, shorten the lifespan of *Drosophila melanogaster*. However, the lethal effects of Aβ are not apparent until after day 15. We used *shibire*^*TS*^ flies that exhibit a temperature-sensitive paralysis phenotype as a reporter of proteostatic robustness. In this model, we found that increasing age but not Aβ expression lowered the flies' permissive temperature, suggesting that Aβ did not exert its lethal effects by proteostatic disruption. Instead, we observed that chemical challenges, in particular oxidative stressors, discriminated clearly between young (robust) and old (sensitive) flies. Using nuclear magnetic resonance spectroscopy in combination with multivariate analysis, we compared water-soluble metabolite profiles at various ages in flies expressing Aβ in their brains. We observed 2 genotype-linked metabolomic signals, the first reported the presence of any Aβ isoform and the second the effects of the lethal Arctic Aβ. Lethality was specifically associated with signs of oxidative respiration dysfunction and oxidative stress.

## 1 Introduction

Many common disorders of the elderly are linked to the deposition of specific-protein aggregates resulting in damage to neurologic, vascular, and endocrine tissues. The incidence of these conditions increases approximately exponentially with age, and consequently, the underlying mechanisms have become the focus of much research (Morimoto, 2008, Taylor and Dillin, 2011). Some of the common pathologic protein aggregates include plaques (Hardy and Selkoe, 2002) and tangles (Mandelkow et al., 2007) in Alzheimer's disease (AD; Blennow et al., 2006), whereas other amyloid deposits are associated with disorders such as amyloid angiopathy (Revesz et al., 2002) and type II diabetes (Moreira, 2012). As the vast majority of these pathologic hallmarks only become apparent in old age, it has been natural to question whether a common cause is a failure of the organism to maintain the correct, native conformation of proteins throughout its life. This concept of “proteostatic collapse” occurring in midlife is supported by in vivo data, particularly from experiments conducted with the nematode worm *Caenorhabditis* *elegans* (reviewed in Balch et al., 2008, Taylor and Dillin, 2011). Studies led by Morimoto and Dillin indicate a concerted loss of stability of a wide range of proteins during midlife in the worm; moreover, this destabilization can be accentuated by the presence of a protein misfolding stressor such as an aggregation-prone poly-glutamine peptide (Ben-Zvi et al., 2009, Gidalevitz et al., 2006).

The underlying mechanisms behind this proteostatic collapse are insufficiently understood. There is evidence for the involvement of genetically regulated responses to, for example, calorie restriction (Kenyon et al., 1993) and heat shock (Morimoto, 1993); however, several other factors may also play a key part in this process. Among these, the role of oxidative stress and age-related metabolic changes may be of particular importance (Finkel and Holbrook, 2000, Houtkooper et al., 2011). It is not clear whether proteostatic collapse causes metabolic changes or vice versa. However, there is strong biochemical evidence showing that long-lived proteins undergo oxidative and other chemical changes that progressively impede their function (Grune et al., 2001, Terman and Brunk, 2006). In addition, the incidence of metabolic disorders such as diabetes also correlates with aging and in many patients diabetes and proteostatic disorders such as AD are both present (Biessels et al., 2006, Munch et al., 1998).

The importance of metabolic alterations as part of the pathology of age-related neurodegenerative disorders such as Alzheimer's has been supported by an array of studies conducted with mouse models and clinical samples from patients with AD (reviewed in Barba et al., 2008, Trushina and Mielke, 2013). Some plausible biomarkers of AD include n-acetylaspartate that reports neuronal loss and myo-inositol which is a marker of gliosis and inflammation. A recent lipidomic study has indicated that a panel of 10 metabolic biomarkers is sufficient to allow confident prediction of which patients with a mild cognitive impairment will progress to AD (Mapstone et al., 2014). Such metabolic markers can be used to detect disease onset and then monitor pathologic progression. Although the metabolic changes in AD are distinct from normal aging, it remains unclear whether these changes are the cause or effect of the associated-protein aggregation pathology.

Defining the processes underpinning the age-related incidence of AD is of practical importance, as diagnostic assays based on genomics, proteomics, and metabolomics are all now possible. By understanding the initial steps that lead to pathology, we can identify biomarkers and develop assays that will allow early diagnosis and then treatment of age-related disorders. In this study, we have used a well-characterized *Drosophila* model (Crowther et al., 2005) as a tool to extend our understanding of how proteostatic deficits and metabolomic changes may lead to the age-related toxicity of the amyloid beta (Aβ) peptide (Rogers et al., 2012).

## 2 Methods

### 2.1 Fly stocks

*Drosophila* cultures were maintained on standard fly medium containing 1.25% (w/v) agar, 10.5% (w/v) dextrose, 10.5% (w/v) maize, and 2.1% (w/v) yeast that were supplemented with a fungicide (methylparaben) and sparse grains of dried yeast on the surface. Fly cultures were maintained in bottles and vials at 18 °C, 25 °C, or 29 °C according to standard *Drosophila* husbandry techniques (Ashburner et al., 2005). Experiments using the inducible gene switch (GS) driver (Osterwalder et al., 2001) for inducible Aβ expression were performed with flies carrying 2 transgenes for Aβ_42_. This stock has been characterized previously (Luheshi et al., 2007). Unless otherwise stated, all other experiments were carried out with single transgenic Aβ_40_, Aβ_42_, and Arctic Aβ_42_ flies which have been described previously (Kruppa et al., 2013). The Aβ transgene was inserted into the 51D location on the second chromosome using the φC31 integrase system (Bischof et al., 2007). Temperature titration experiments to measure the protein homeostasis were performed with *Drosophila shibire*^*TS2*^ constructs, which have been described earlier (Grigliatti et al., 1973).

### 2.2 Longevity assays with constitutive Aβ expression

Longevity assays were conducted as described previously (Crowther et al., 2005). *Drosophila* stocks carrying various UAS-Aβ isoforms were crossed with the *elav*^*c155*^-Gal4 promoter stock to induce pan-neuronal transgene expression in the offspring. Longevity experiments were performed on populations of 90–100 mated females, sorted into groups of 10. The statistical interpretation of longevity data was conservative: we considered the median survival values for each of the 9–10 subgroups of flies per condition as estimates of the population mean. According to, the Wilcoxon-rank test was used to determine whether the median survivals of the population pairs were significantly different from each other. When estimating mortality trajectories; a minimum of 150 flies per genotype were assessed. Mortality trajectories were generated by plotting the natural log of the chance of dying over the coming 24 hours versus the age of the flies. The first and last 5% of all recorded death events were excluded to minimize noise as described previously (Shaw et al., 1999).

#### 2.2.1 Longevity assays with inducible Aβ expression

The mifepristone (RU 486, Sigma-Aldrich) inducing agent was dissolved at 10 mg/mL in ethanol and further diluted in sterile water to its final concentration. Vials of fly food were treated with 300 μL of the drug solution, or vehicle, and left to air dry for 2 days at room temperature (25 °C) before use. The food was used within 4 days of preparation or else discarded. To achieve inducible, pan-neuronal, expression of Aβ with the GS system, *Drosophila* homozygous for the Aβ_42_ transgene on the second and third chromosomes (Aβ_42.3_; Aβ_42.4_, as previously described in [Crowther et al., 2005]) were first crossed with the *GS-elav-Gal4* driver line and the offspring treated with 200 μg/mL of RU 486. In other respects, longevity assays using drug-inducible Aβ expression (Osterwalder et al., 2001), were identical to the protocol for constitutive Aβ expression as described previously (Crowther et al., 2005).

#### 2.2.2 Temperature titration locomotion assays with Drosophila shibire^TS2^ mutants

The effect of Aβ expression on the shibire^*TS2*^-mediated paralytic phenotype was assessed for flies of various ages. *UAS-Aβ* stocks were crossed with flies carrying the constitutive pan-neuronal *elav-Gal4* driver (chromosome 3). Male offspring carrying both transgenes, over balancer chromosomes, were mated with females homozygous for the *shibire*^*TS2*^ allele (X chromosome). The walking velocities of male offspring carrying *shibire*^*TS2*^ ± Aβ expression were recorded by using the iFly tracking system described previously (Jahn et al., 2011). The climbing velocity of 30 male flies, in 3 groups of 10 was assessed in triplicate for each genotype at day 5, 15, and 35 after eclosion. All *Drosophila shibire*^*TS2*^ mutants were maintained and aged at the permissive temperature of 18 °C. Before each measurement, the flies were incubated for 90 seconds in preheated glass vials in a water bath at required temperature. Differences in climbing performance between genotypes were probed for statistical significance by a 2-way analysis of variance (ANOVA) using the GraphPad Prism statistical package (Graph Pad Prism Software, Inc.).

### 2.3 Hydrogen peroxide lethality assays

Mated female flies were sorted into at least 8 groups of 20 and cultured at 25 °C throughout the experiment. The control population was maintained on 2% (w/v) agar containing 5% (w/v) sucrose. The food was supplemented with 10% (v/v) hydrogen peroxide for the test population. Survival rates 72 hours postexposure were tabulated. The statistical significance of mortality differences between genotypes throughout aging was assessed by a 2-way ANOVA.

### 2.4 Brain dissections

Brain dissections were performed as described previously (Costa et al., 2011).

### 2.5 Quantitative polymerase chain reaction

Quantitative polymerase chain reaction for Aβ mRNA was carried out as described previously (Kruppa et al., 2013).

### 2.6 Quantification of Aβ

The procedure has been described elsewhere (Luheshi et al., 2010). For the quantification of total Aβ levels, heads from 10 flies were collected and homogenized in 20 μL 5 M guanidine hydrochloride (GnHCl; Melford Laboratories, UK) extraction buffer (1x stock: 50 mM Hepes pH 7.3, 5 mM ethylenediaminetetraacetic acid, and 5 M GnHCl) manually for 1 minute. The samples were then sonicated in a water bath at room temperature for 480 seconds and centrifuged for 7 minutes at 18,000 g. Five μL of supernatant were diluted down 1:5 in a Meso Scale Discovery (MSD) dilution buffer (50 mM Hepes pH 7.3, 10 mM ethylenediaminetetraacetic acid, 0.1% (w/v) MSD Blocker A [bovine serum albumin in a phosphate buffered saline (PBS)-based buffer]; MSD, USA). Each 96 well Avidin or Streptavidin plate (MSD, USA) was precoated with 3% (w/v) MSD-Blocker A in PBS either for 1 hour at room temperature on a shaker or overnight at 4 °C. Between each of the next steps, the plate was washed with 0.05% (v/v) PBS-Tween. Samples were incubated for 1 hour at room temperature with the biotinylated 6E10 (Cambridge Biosciences, UK) primary antibody (epitope located within the 3-8 N-terminal amino acids of Aβ), followed by 1.5 hours of incubation with the calibration and test samples in the various wells (the standard curve mixture, water, and the *Drosophila* samples). At last, 1 hour incubation at room temperature with the sulpho-tagged 21F12 secondary antibody (epitope located within the 33-42 C-terminal amino acids of Aβ, Elan Pharmaceuticals, USA) was performed. The plate was read on an MSD-plate reader (MSD, USA) according to the manufacturer's instructions. For measuring soluble Aβ 1% (w/v) sodium dodecyl sulfate (SDS) in PBS buffer was used for the initial extraction instead of 5 M GnHCl. The remaining steps of the protocol were identical except for the centrifugation step: a 7 seconds pulse spin was used instead, to ensure the peptide remained in the supernatant. The standard curve for the assay was constructed using the SDS-extraction protocol and was used to estimate concentrations for both SDS-soluble and total Aβ assays; this may result in significant underestimation of total Aβ levels because of GnHCl interference in the latter assay. Differences in Aβ concentrations between samples were probed by the Student's *t*-test and the ANOVA Bonferroni test.

### 2.7 ^1^H-Nuclear magnetic resonance (NMR) spectroscopy

To induce pan-neuronal expression of Aβ in the progeny, *UAS-Aβ*_*40*_, *UAS-Aβ*_*42*_, and *UAS-Aβ*_*42*_ *Arctic* stocks were crossed with the *elav*^*c155*^*-Gal4* promoter. To control for metabolic changes in the absence of Aβ expression, *elav*^*c155*^ flies were crossed with a stock containing the empty 51D insertion site. Subsequently, the progeny of this stock are referred to as “51D control” in the text. The offspring with the desired genotype were aged at 29 °C before harvesting. Flies were collected according to a computer-generated random scheme that avoided any time-of-day or position-in-cohort artefacts. Once collected, the flies were frozen in liquid nitrogen and decapitated by vortexing while frozen. Fly heads and bodies were homogenized separately in 350 μL of ice-cold 50% (v/v) acetonitrile (Merck, Germany) in distilled water solution by using an electric homogenizer. The samples were then centrifuged for 20 minutes at 18,000 g (Minispin centrifuge; Eppendorf, Germany) at 4 °C, and the supernatant was transferred into a new microcentrifuge tube, lyophilized, and stored at −80 °C. Each sample consisted of either 50 fly heads or bodies. The experiment was performed in triplicate per condition. Before NMR analysis, the samples were rehydrated in 650 μL of 50 mM phosphate buffer in D_2_O (pH 7.4) supplemented with 50 mg/L 3-trimethylsilyl propionic acid D4 (TSP) as a chemical shift reference, and 50 mg/L sodium azide to avoid bacterial growth. Subsequently, 600 μL of the rehydrated sample were transferred into a 5-mm NMR tube. The NMR measurements were carried out at 25 °C on a Bruker Avance-III 600 spectrometer (Bruker Biospin, Germany) which was equipped with a double-tuned ^1^H-^13^ C cryoprobe and operated at a ^1^H frequency of 600.13 MHz. The ^1^H NMR spectra were acquired using a single 90° pulse experiment with a Carr Purcell Meiboom Gill delay added, to attenuate broad signals from high molecular weight components. The total Carr Purcell Meiboom Gill delay was 40 ms and the spin echo delay 200 μs. The water signal was suppressed by presaturation of the water peak during the relaxation delay of 4 seconds. A total of 98,304 data points spanning a spectral width of 24 ppm were collected in 64 and 128 transients for fly bodies and heads, respectively. For assignment purposes, 2-dimensional ^1^H-^1^H COSY, ^1^H-^1^H TOCSY, ^1^H-^13^ C HSQC, ^1^H-^13^ C HSQC-TOCSY, and ^1^H-^13^ C HMBC spectra were acquired. The spectra were processed using iNMR (www.inmr.net). An exponential line broadening of 0.5 Hz was applied to the free induction decay, before Fourier transformation. All spectra were referenced to the TSP signal at −0.017 ppm, automatically phased and baseline corrected. The spectra were aligned using Icoshift (Savorani et al., 2010), and the region around the residual water signal (4.88–4.67 ppm) was removed. Moreover, the high- and low-field ends of the spectrum, where no signals except the reference signal from TSP appeared, were also removed (i.e., leaving data between 9.5 and 0.5 ppm). The integrals were normalized to total intensity to suppress trivial separation based on variations in sample amount, and the data were scaled using pareto scaling (Craig et al., 2006) and centered.

#### 2.7.1 NMR data analysis

Initially, the whole data set was subjected to principal component analysis (PCA) (Stoyanova and Brown, 2001). Afterward, orthogonal projection to latent structures discriminant analysis (OPLS-DA) models were created to separate the following 3 classes of fly populations: (1) control flies, (2) Aβ_42_ expressing flies that showed a minimal decrease in longevity under the tested conditions, and (3) Arctic Aβ_42_ flies which showed a significant decrease in median survival in a longevity assessment that was run in parallel. OPLS-DA models are multivariate models that predict group membership based on a multivariate input, in this case the NMR spectra. The model separates variations because of group membership from other (orthogonal) variations (Trygg and Wold, 2003). The models were constructed by using the spectral data of 18- and 21-day-old flies and were then implemented to predict the class and age for all other flies. To identify metabolites that responded to nontoxic Aβ expression, we constructed OPLS-DA models using data from 14- to 24-day-old 51D controls that do not express Aβ, and compared them to Aβ_40_ and Aβ_42_ flies where Aβ expression was largely nontoxic. Similarly, to probe for metabolites associated with Aβ *toxicity*, we compared profiles of Aβ_40_ and Aβ_42_ flies with those from flies expressing the lethal Arctic Aβ_42_. The loadings and the correlation coefficient (*R*) between intensities at the individual frequencies and the predictive component were calculated. A cutoff value for *R*^2^ corresponding to *p* < 0.01 with Bonferroni correction for an assumed number of 100 metabolites was calculated from the distribution of *R*^2^ values in 10,000 permutated data sets. Xanthurenic acid was confirmed by spiking. The remaining assignments were done based on chemical shifts only, using earlier assignments, and spectral databases described elsewhere (Cui et al., 2008, Malmendal et al., 2006, Pedersen et al., 2008) and comparison with *Drosophila* metabolites identified by mass spectrometry (Chintapalli et al., 2013). OPLS models predict the value of a continuous variable based on a multivariate input (Trygg and Wold, 2002). OPLS models for age were built using 14 to 40-day-old control and Aβ_42_ flies. These models were used to predict the “metabolomic age” of samples for Aβ_40_ and Arctic Aβ_42_ expressing *Drosophila*. All multivariate analysis was performed using the Simca-P software (Umetrics, Sweden). All procedures were done for both fly heads and bodies.

## 3 Results

### 3.1 Aging increases the sensitivity of *Drosophila* to the toxic effects of Aβ

**Supplementary Fig. 1:**
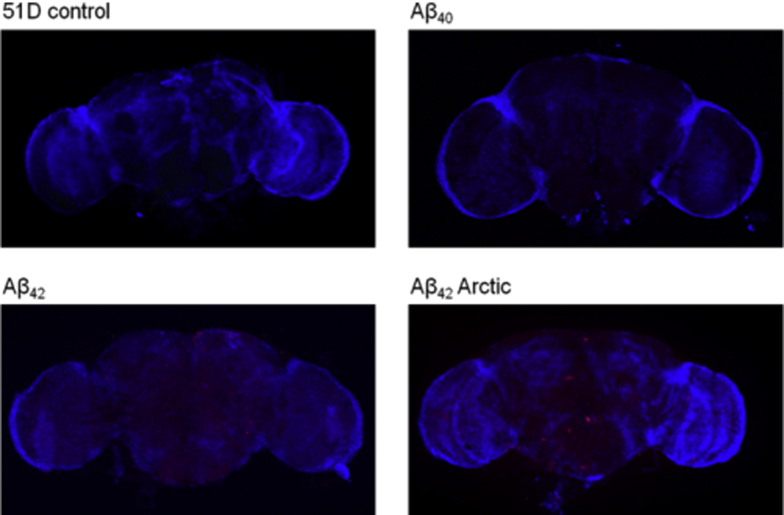


**Supplementary Fig. 2:**
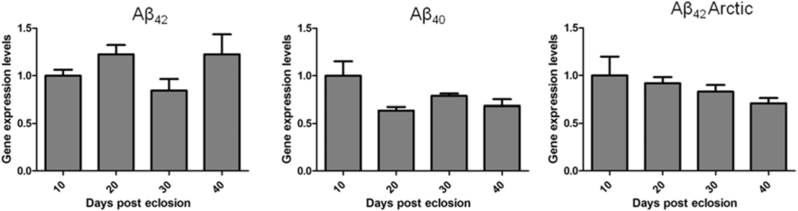


**Fig. 1:**
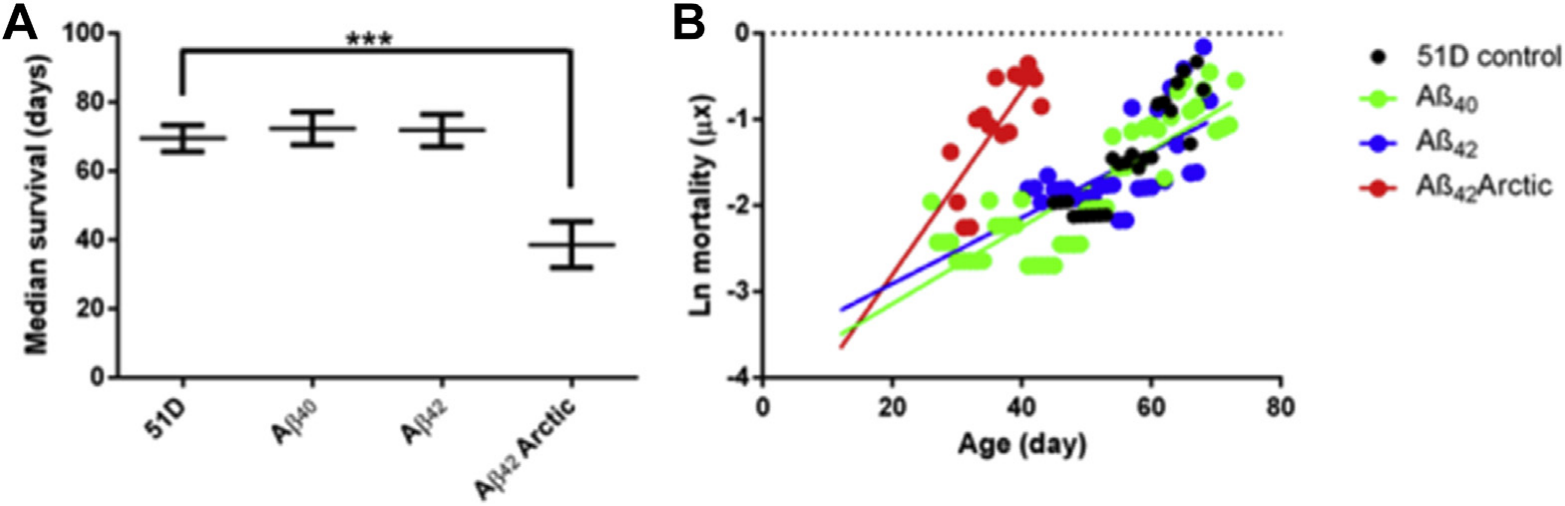
The expression of Aβ42 Arctic but not Aβ42 or Aβ40 reduces median survival. (A) Pan-neuronal expression of Aβ40 or Aβ42 did not decrease the median survival of flies as compared to the 51D control population that does not express Aβ. The Arctic isoform of Aβ42 was toxic and approximately halved the median survival. Differences in median survivals were assessed by the Mann–Whitney test (*** *p* < 0.001, error bars show the standard deviation). (B) A plot of the natural log of the daily chance of dying indicates that the linear mortality trajectory for flies expressing Arctic Aβ42 appears to diverge from flies expressing other A isoforms at approximately day 15. These experiments were conducted at 25 °C with at least 15 groups of 10 flies. Abbreviation: Aβ, amyloid beta.

The toxicity of Aβ peptides in our *Drosophila* model system is dependent on both the aggregation propensity of each isoform and the intensity of its expression. When cultured at 25 °C, flies carrying Aβ_40_ and Aβ_42_ transgenes inserted at the 51D genomic locus and driven by *elav*^*c155*^*-Gal4* exhibit no reduction in longevity (Fig. 1A) and do not show Aβ deposition (Supplementary Fig. 1). By contrast, flies expressing the aggregation prone Arctic (E22 G) variant (Nilsberth et al., 2001) of Aβ_42_ under the same conditions have a shorter lifespan and display Aβ deposits in the brain (Fig. 1A and Supplementary Fig. 1). These fly lines have been extensively characterized, and the transgenes have been shown to express mRNA at equivalent levels (Crowther et al., 2005). Moreover, we also confirmed that Aβ-mRNA levels did not change significantly throughout life (Supplementary Fig. 2).

When we calculated the mortality trajectories (logarithm of the daily probability of death), we found that the 51D control, Aβ_40_ and Aβ_42_ lines followed the same linear trajectory, reflecting the expected exponential increase in the likelihood of death with age. The equivalent trajectory for Arctic Aβ_42_ flies was also linear but was steeper, indicating that the age-related probability of death in these flies was rising more rapidly than for flies expressing Aβ_40_ and Aβ_42_. Remarkably, when the linear fits were extrapolated back in time, the Arctic Aβ_42_ flies did not diverge from the other lines until a point in midadult life around day 15 (Fig. 1B).

**Fig. 2:**
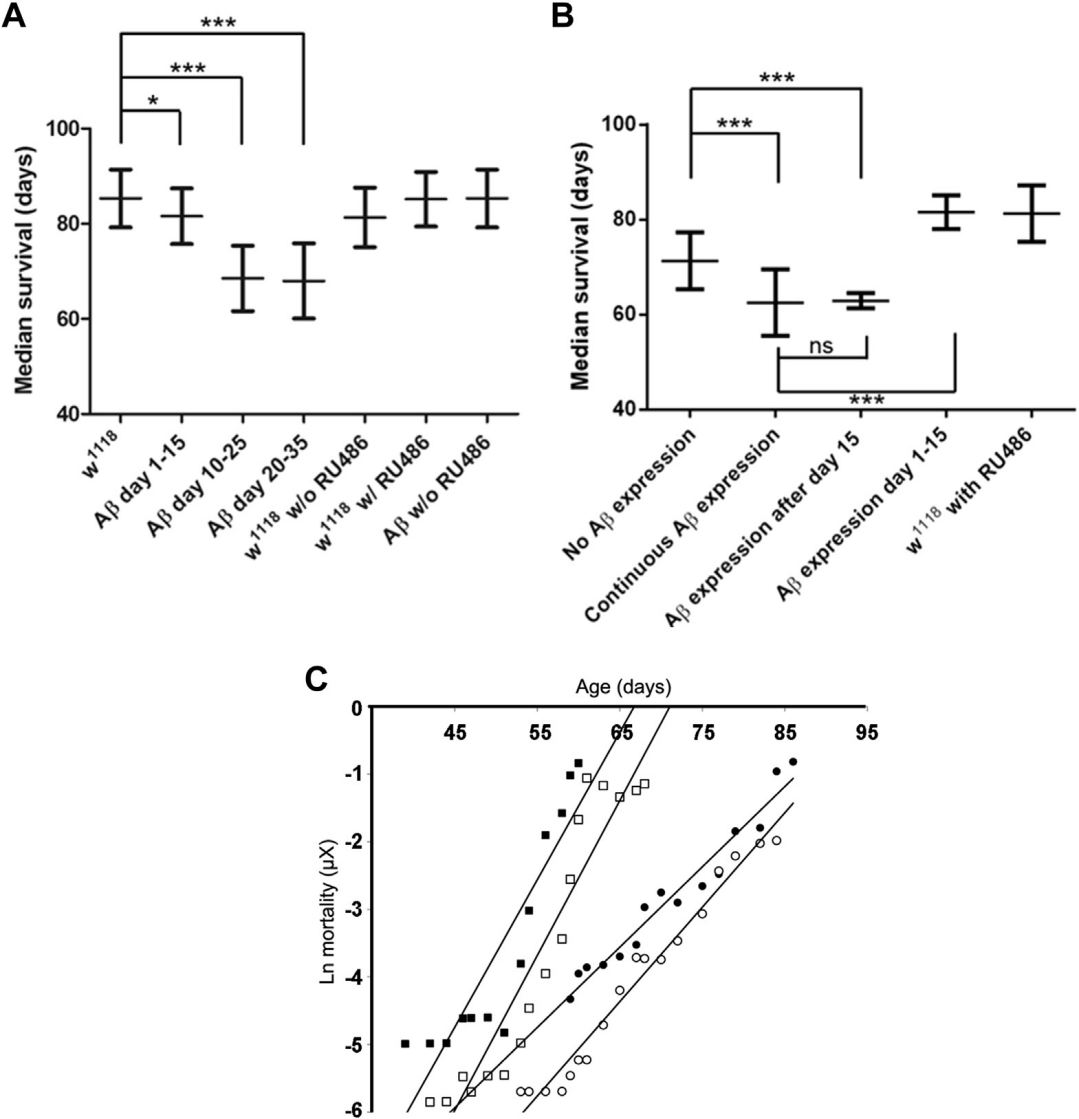
Young flies are resistant to the toxic effects of Aβ expression. (A) Aβ42 expression in early adulthood (days 1–15) resulted in minimal reduction in longevity, however, later windows of expression (days 10–25 and 20–35) resulted in larger, approximately 20-day reductions in median survival. The RU486 induction agent had no effect on the median survival of control flies (*w1118*). (B) As compared with control flies (“no A”), where Aβ is never induced, continuous expression of Aβ reduced longevity. Flies in which Aβ expression were limited to days 1–15 had a lifespan that was comparable with controls, whereas expression of Aβ from day 15 onward led to a median survival essentially equivalent to continuous Aβ exposure. Flies were cultured at 25 °C in 10 groups of 10 flies. Differences in median survivals were assessed by the Mann–Whitney test (* *p* < 0.05, *** *p* < 0.001, error bars show the standard deviation). (C) Mortality trajectory analysis of the flies in B indicate that Aβ expression from day 1–15 results in an upward displacement of the mortality trajectories by between 1.2 (closed vs. open squares) and 0.7 (closed vs. open circles; see also Supplementary Table 1). In adult flies, Aβ expression increased the gradient of the mortality trajectories by between 0.90 day 1 (Fig. 2C, “Aβ day 15+” vs. “no Aβ”) and 0.10 day 1 (Fig. 2C, “continuous Aβ” vs. “Aβ day 1–15”) from day 35 onward. In these experiments, flies carried 2 transgenes for Aβ42. Abbreviations: Aβ, amyloid beta; ns, not significant.

Thus, young flies (<15 days) appeared more robust and showed less marked increases in the likelihood of death in the presence of Aβ isoforms that are otherwise lethal when present later in life. To investigate this observation directly, we set up a series of flies that carried two Aβ_42_ transgenes under the control of an inducible promoter. Our initial experiments examined the longevity effects of 15-day windows of Aβ induction, specifically days 0–15, 10–25, and 20–35 (Fig. 2A). Here, we did not observe any substantial reductions in longevity when Aβ expression was triggered only before day 15. However, when Aβ expression was induced after this age, we recorded significant reductions in lifespan.

**Supplementary Fig. 3:**
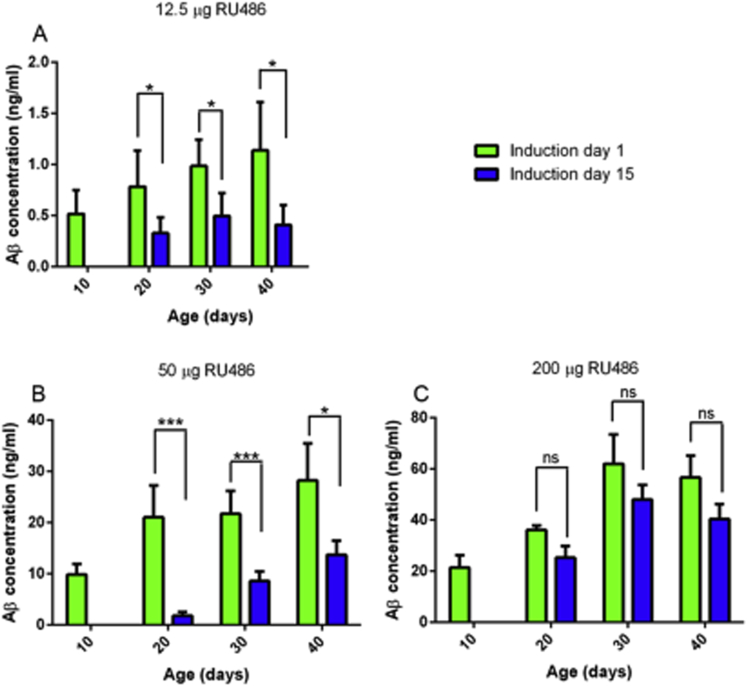


A subsequent experiment compared the survival of 4 groups of *Drosophila*: (1) where Aβ expression was never induced, (2) where Aβ was continuously induced, (3) where Aβ expression was induced from hatching until day 15, and (4) where Aβ was induced only after day 15 (Fig. 2B). To ensure that the levels of expressed Aβ were similar for all groups, we undertook a series of experiments in parallel to calibrate the induction of Aβ expression for all conditions (Supplementary Fig. 3). When we examined the median survivals of the groups we observed that, as expected, flies exposed to continuous Aβ induction had a shorter lifespan than those in which Aβ expression was never induced. Interestingly, restricting the expression of Aβ to midadult life and later (after day 15) resulted in a median survival that was essentially identical to the median survival of flies where Aβ was expressed continuously. On the other hand, when Aβ was expressed only up to day 15, the lifespan of flies was similar to controls with no Aβ induction at all. Taken together, these survival data support the hypothesis that older flies are more sensitive to the lethal effects of Aβ expression as compared with younger flies.

Using mortality trajectory analysis, we were able to estimate this relative robustness of young flies. Specifically, in Fig. 2C the filled squares represent the mortality of flies with continuous Aβ expression; by comparison the open squares represent flies that are identical except that they did not experience Aβ expression from days 0–15. These traces are essentially parallel and are separated on average by a mortality displacement of 1.2. Assuming that this relative displacement accrued linearly over days 0–15 this corresponds to an Aβ-induced increase in the gradient of the mortality curve of 0.08 day^−1^ (standard error of mean: 9 × 10^−3^ day^−1^; see Supplementary Table 1A). For filled circles, the only Aβ expression is from days 0 to 15, and these are compared with empty circles where Aβ was never expressed. Where paired data points are available, the average mortality displacement between these traces is 0.7, resulting in a calculated daily rate of mortality increase of 0.05 day^−1^ (standard error of mean: 8 × 10^−3^ day^−1^) over days 0–15. The average of these 2 estimates of the Aβ-attributable increase in the mortality trajectory gradient between days 0–15 is 0.06 day^−1^.

By contrast, the daily increase in mortality that is attributable to Aβ expression in mature adults can be quantified directly by comparing the gradients of the following paired traces: filled squares versus filled circles (difference in gradients 0.10 day^−1^) and empty squares versus empty circles (difference in gradients 0.09 day^−1^; see Supplementary Table 1B). The average of these 2 estimates of the Aβ-attributable increase in the mortality trajectory gradient days 15+ is 0.10 day^−1^. This allows us to estimate that the Aβ-attributable increase in the mortality trajectory gradient is approximately 50% greater in older adult flies (days 45–85), as compared with young adults (days 0–15).

### 3.2 Aging alters Aβ metabolism, favoring its insoluble deposition

**Fig. 3:**
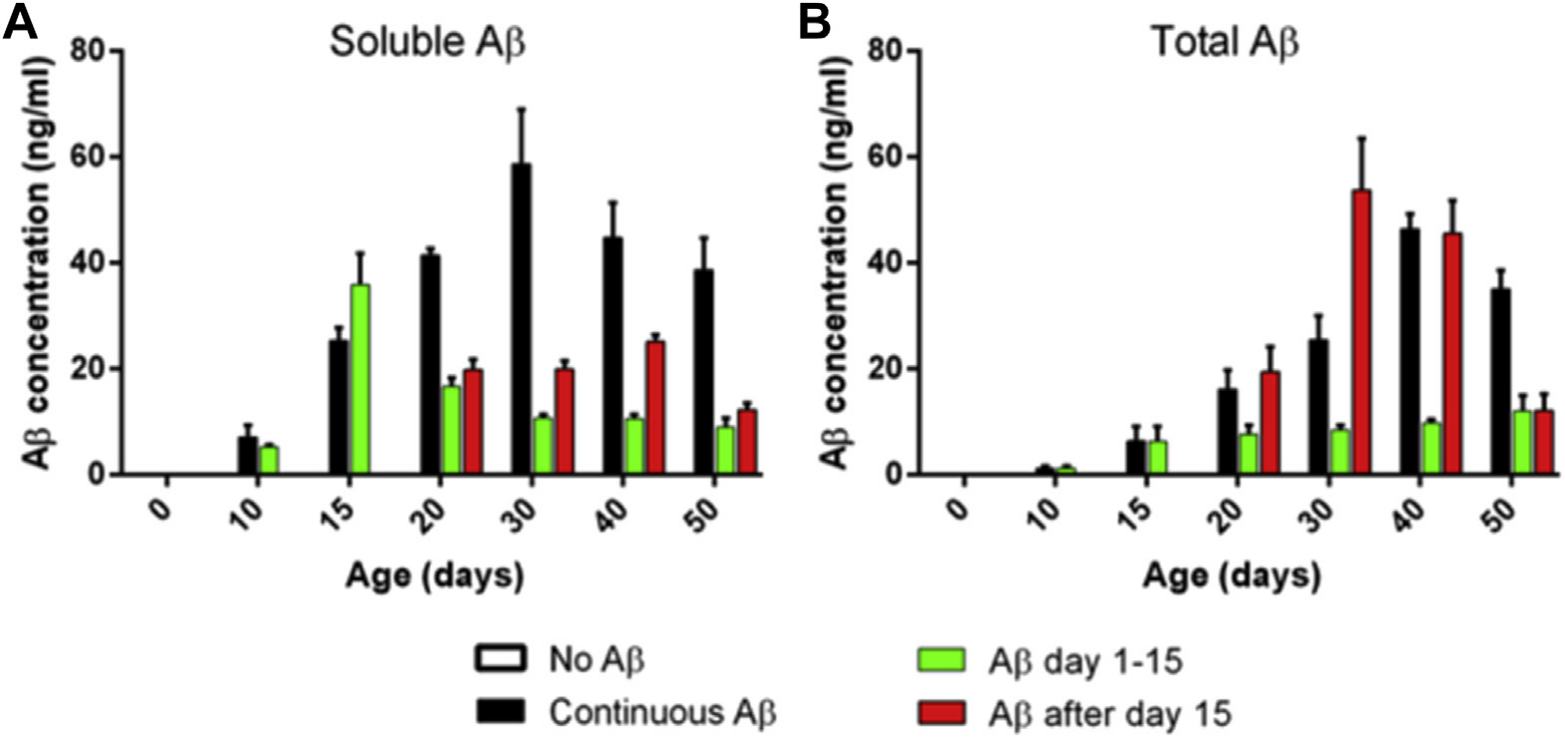
Accumulation of Aβ in fly heads throughout aging. The levels of soluble Aβ42 (panel A; extracted with 1% w/v SDS in PBS) and total Aβ42 (panel B; extracted with 5 M GnHCl) were measured as the flies aged. The levels in the absence of Aβ42 induction (baseline reading) were compared with flies induced continually (black), for days 1–15 (green) and from day 15 onward (red). Error bars show the standard deviation of 3 biological replicates. GnHCl interference with the assay may result in under-estimation of total A levels in panel B, however, comparisons within panel B are valid. Abbreviation: Aβ, amyloid beta; SDS, sodium dodecyl sulfate.

Using inducible pan-neuronal Aβ expression, we compared the accumulation of soluble and total peptide in young and aged *Drosophila* (Osterwalder et al., 2001). One percent w/v SDS extraction yielded soluble peptide, and these levels were compared with total Aβ as defined by GnHCl-extracted material. We observed an initial accumulation of soluble peptide in flies expressing Aβ from days 1–15, which dropped to a base level after the inducing agent was withdrawn (Fig. 3, green). Under such circumstances, there was little increase in total Aβ levels, indicating that the soluble material was largely cleared. When induction was continued, then soluble and subsequently insoluble peptide progressively accumulated (30–40 days, black). Induction of Aβ exclusively in the older flies (red, day 15+), yielded relatively little soluble material with a higher proportion accumulating as insoluble deposits rather than being cleared.

### 3.3 Aβ toxicity is not accompanied by a generalized protein homeostasis collapse

**Fig. 4:**
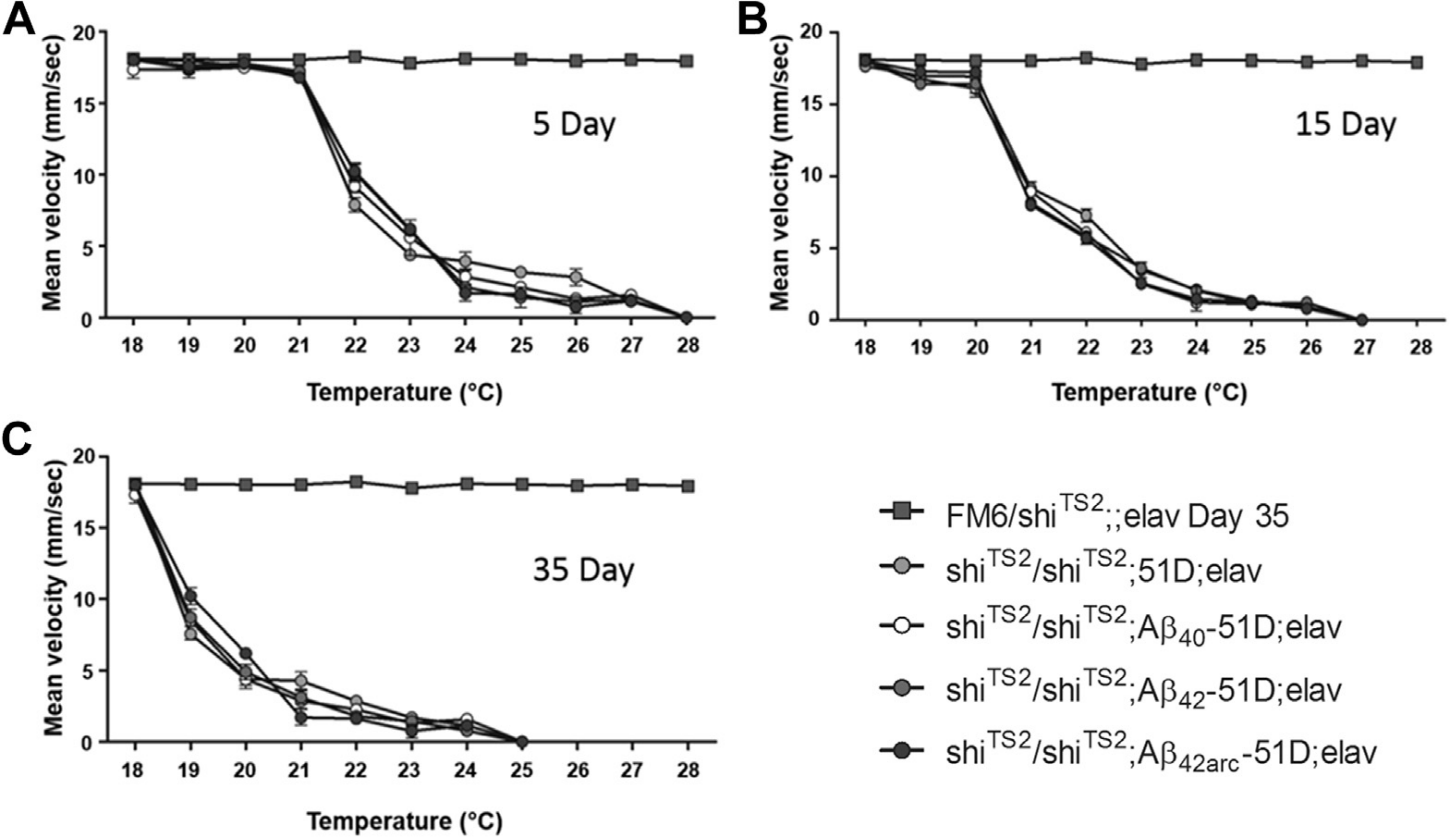
Aβ coexpression does not exacerbate the age-related heat sensitivity of *Drosophila shibireTS2*. Titration of temperature with climbing performance of *shibireTS2* flies at different ages demonstrated that although *Drosophila* homozygous for *shibireTS2* became paralyzed at increasingly lower temperatures as they aged (panels A through to C), the coexpression of Aβ did not exacerbate this paralytic phenotype. The locomotor assessment was carried out with 3 biological replicates per condition. The video assessment of walking velocity was undertaken as described previously (Jahn et al., 2011). A 2-way ANOVA analysis of the climbing performance did not show any differences between the genotypes. The flies were crossed and maintained at 18 °C before each locomotor assessment. The 1x *shibireTS2* negative control data (rectangles) for all panels represents the behavior of the oldest day 35 flies. Abbreviations: ANOVA, analysis of variance; Aβ, amyloid beta.

Previous studies, particularly in the nematode worm *C*. *elegans*, have indicated that a concerted loss of protein homeostasis accompanies the onset of protein misfolding and amyloid deposition. It was also shown that additional misfolding stressors can destabilize the proteome (Ben-Zvi et al., 2009, Gidalevitz et al., 2006). To test whether Aβ expression precipitates proteostatic collapse in *Drosophila*, we assessed the locomotor behavior of a temperature-sensitive dynamin variant (*shibire*^*TS2*^) using paralysis as a reporter of proteome stability (van der Bliek and Meyerowitz, 1991). We have developed a novel assay in which *shibire*^*TS2*^ flies expressing various isoforms of Aβ, or not, undergo a temperature titration to determine how sensitive they are to thermal stress (Fig. 4). In all tested *Drosophila*, we observed an age-related increase in temperature sensitivity, specifically the temperature at which the flies walked at half their normal velocity, was reduced from 22 °C at day 5 °C–19 °C at day 35. Similarly, the temperature at which the flies were all paralyzed was reduced from 28 °C at day 5 °C–25 °C at day 35.

**Supplementary Fig. 4:**
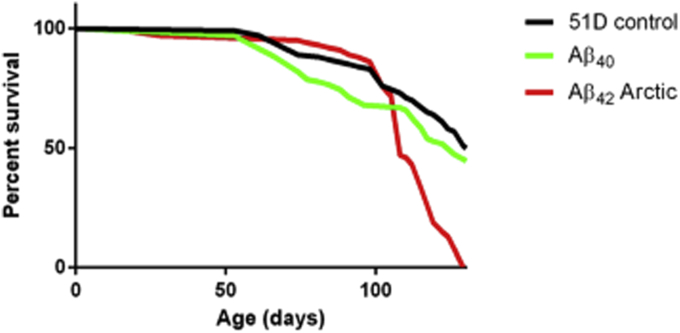


These data indicate that the *shibire*^*TS2*^ variant of dynamin becomes thermally destabilized as the flies age. However, this effect was not modified by Aβ-peptide expression, including the Arctic isoform that is toxic under these conditions (Supplementary Fig. 4). The failure of Aβ expression to modulate the age-related thermal destabilization of dynamin is consistent with the hypothesis that accelerated proteostatic collapse is not the primary mechanism of Aβ toxicity in our model system.

### 3.4 Aging and Aβ expression predispose *Drosophila* to oxidative damage

**Fig. 5:**
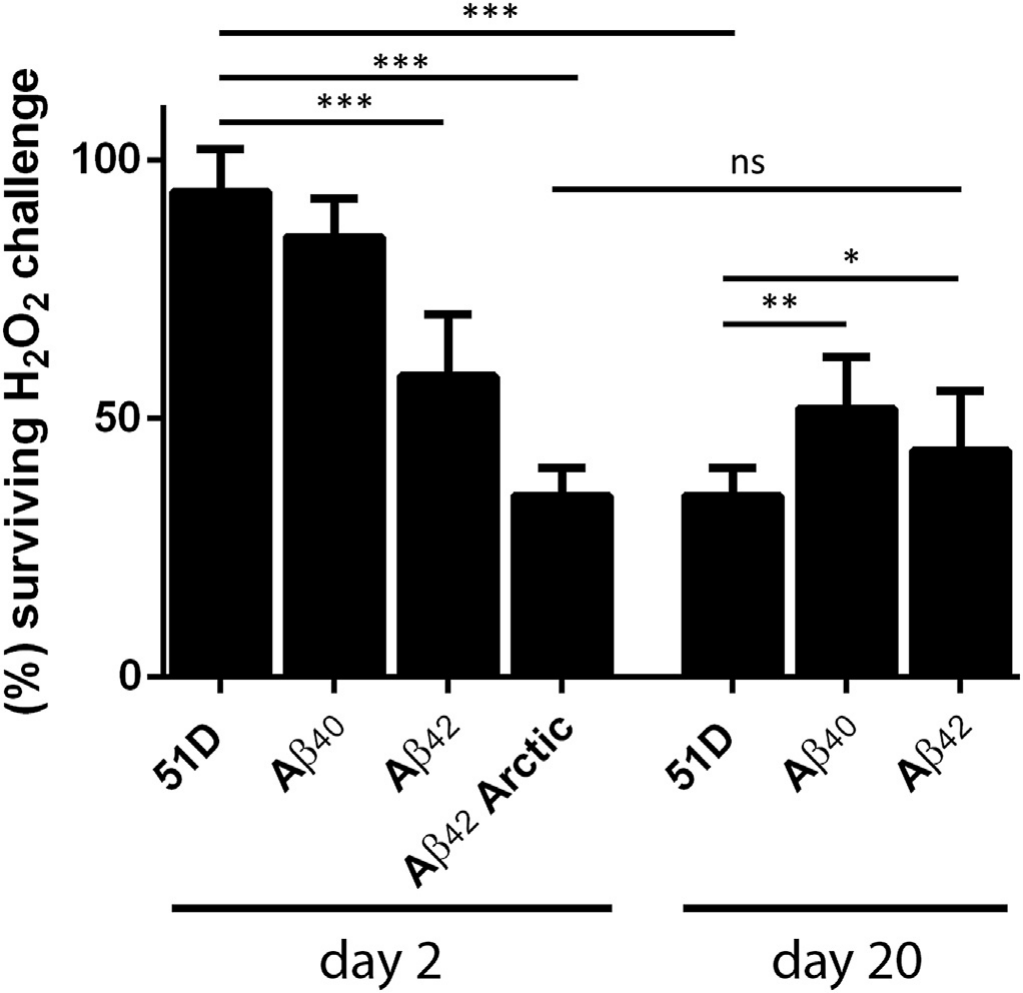
Aging and Aβ expression increase the sensitivity of *Drosophila* to oxidative stress. Young (day 2) control flies, and those expressing Aβ40, were relatively resistant to 72 hours of hydrogen peroxide treatment. All older flies (day 20) were more sensitive to this oxidative challenge. Remarkably, young flies expressing the aggregation-prone Aβ42 isoforms were as sensitive to oxidative stress as the old flies. No deaths were observed in the control experiment where the flies were kept on equivalent food without hydrogen peroxide (data not shown). The statistical significance of mortality differences between genotypes with age was assessed by 2-way ANOVA (* *p* < 0.05, ** *p* < 0.01, *** *p* < 0.001, error bars show the standard deviation). Abbreviations: ANOVA, analysis of variance; Aβ, amyloid beta; ns, not significant.

Age-related changes in metabolism, in particular the generation of reactive oxygen species, are an alternative factor that may mediate the increased sensitivity of older flies to Aβ toxicity (Finkel and Holbrook, 2000, Rival et al., 2009). To test this possibility, we gave an oxidative stressor, in the form of 10% (v/v) H_2_O_2_ in the food, to both young and older flies. At day 2, control flies and those expressing Aβ_40_ were remarkably resistant to this insult with few dying after 72 hours of exposure. By contrast, the young Aβ_42_ expressing flies were more sensitive with approximately half dying after the same treatment (Fig. 5, left bars). Lethality was even higher for Aβ_42_ Arctic flies. In this regard, the day 2 Aβ_42_ flies resemble older, day 20 flies, which uniformly exhibit increased sensitivity to H_2_O_2_ regardless of Aβ expression (Fig. 5, right bars). Thus, day 2 flies are proteostatically robust but sensitive to oxidative stress when they express Aβ_42_, putting the biochemical changes pathologically upstream of proteome dysfunction.

### 3.5 A systematic analysis of the metabolic consequences of Aβ expression in the *Drosophila* brain

**Fig. 6:**
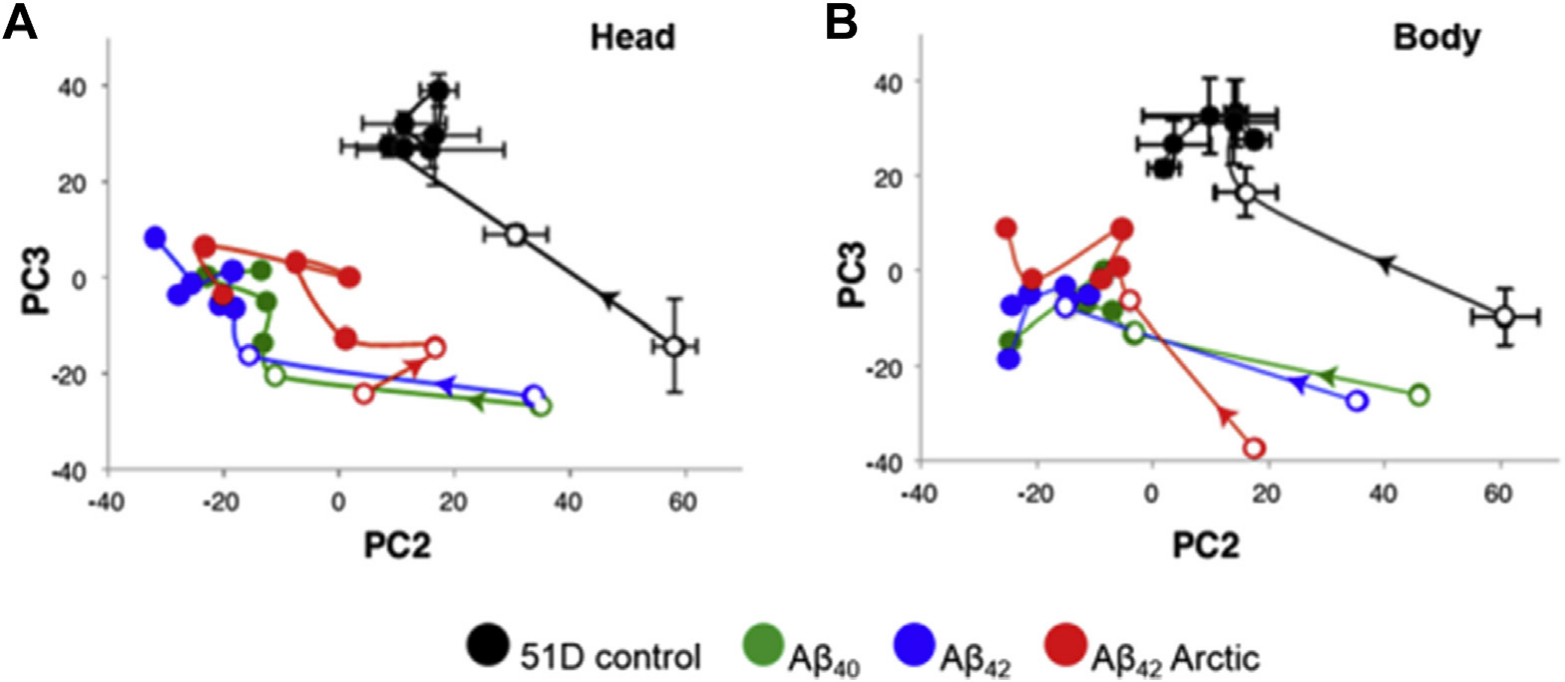
The expression of Aβ causes a shift in the age-related changes in fly metabolomes. The figure shows score plots for principal components 2 and 3 for all head (A) and body (B) samples. The metabolomic trajectories are indicated by colored lines (open circles for days 0 and 8; closed circles for days 14, 18, 21, 24, 30, and 40; A42 Arctic flies (red) lived only until day 26 which is their last sampling point). For clarity, error bars showing the standard deviation are presented for the 51D control flies only. The error bars for all other points were of a similar magnitude. Abbreviation: Aβ, amyloid beta.

To assess the age-related changes in the metabolome in control flies and those expressing Aβ, we have compared the water-soluble metabolite levels in tissue extracts by ^1^H-NMR spectroscopy. Flies were aged and at specified times frozen and separated into heads and bodies so that the local and systemic effects of Aβ expression could be assessed. Our initial approach was to analyze the NMR spectra using PCA. When we plotted principal component 2 (PC2) versus PC3 it became apparent that there was an age-related trajectory that all the samples were following (Fig. 6). In almost all cases, the greatest differences were between days 1 and 8 (open circles), and these were broadly conserved between genotypes. Moreover, the expression of any Aβ isoform produced a displacement of the trajectories as compared with the scores of 51D control samples. This indicates the presence of profound metabolic changes around the time of hatching in addition to those that can be attributed to Aβ expression. PC1 reported metabolic changes that did not correlate with genotype or age and was not analyzed further. Furthermore, a 2-way multivariate analysis of variance on all PCs revealed significant (*p* < 1×10^−15^) differences in the age-linked metabolomic profiles for 51D controls versus Aβ_40_/Aβ_42_ (controls vs. nontoxic Aβ expression) and for Aβ_40_/Aβ_42_ versus Arctic Aβ_42_ (nontoxic Aβ vs. toxic Aβ expression), for both bodies and heads.

### 3.6 Aβ expression induces a distinct metabolite response

**Fig. 7:**
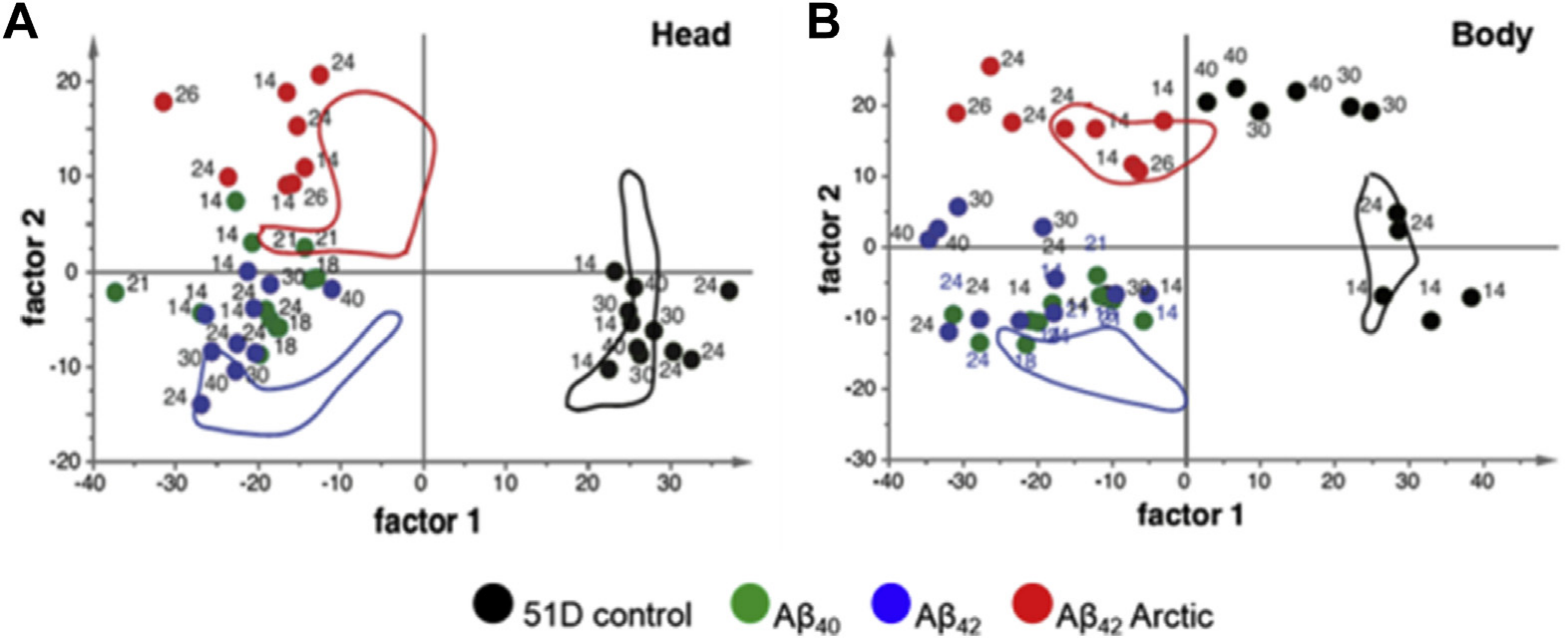
O2PLS-DA modeling reveals 2 independent metabolomic changes, associated with A expression and lethality. O2PLS-DA models were created for head (A) and body (B) samples from mature flies (days 18 and 21) to assign the remaining samples into 3 groups: (1) control flies, (2) Aβ42 expressing flies, and (3) Arctic Aβ42. The digits associated with each data point report the age in days of the flies. The cross-validated scores of the 18- and 21-day-old samples that were used to build the O2PLS-DA model are indicated by the area encompassed by colored lines. Abbreviation: Aβ, amyloid beta.

After day 14, the fly metabolome becomes less temporally dynamic, and so, we created an OPLS-DA model implementing data from mature flies. The goal was to discriminate metabolite profiles between (1) 51D control flies (Fig. 7, black) and those expressing, (2) Aβ_42_ which is aggregation prone but remains nontoxic under the experimental conditions (blue), and (3) Arctic Aβ_42_ which is both aggregation-prone and toxic (red). Accordingly, the model was trained using data from days 18 to 21 flies only; furthermore data from flies expressing Aβ_40_, which has a low-aggregation propensity and is nontoxic (green), were not included. When the model was applied to samples that were not part of the training data set 3 groupings became apparent. First, we observed that the expression of any Aβ isoform resulted in a shift away from controls along the x-axis; this may be interpreted as an adaptive response to aggregation prone peptide expression as this signal is compatible with normal longevity. Among the Aβ-expressing group, there was an additional separation that was associated with reduced longevity as seen in Arctic Aβ flies (Fig. 7, panels A and B). Notably, flies expressing Aβ_40_ were located in the same region as those expressing Aβ_42_. Incidentally, the oldest (30–40 days) control and Aβ_42_ flies became more similar to the Aβ_42_ Arctic flies but only in the systemic metabolome (Fig. 7B).

**Supplementary Fig. 5:**
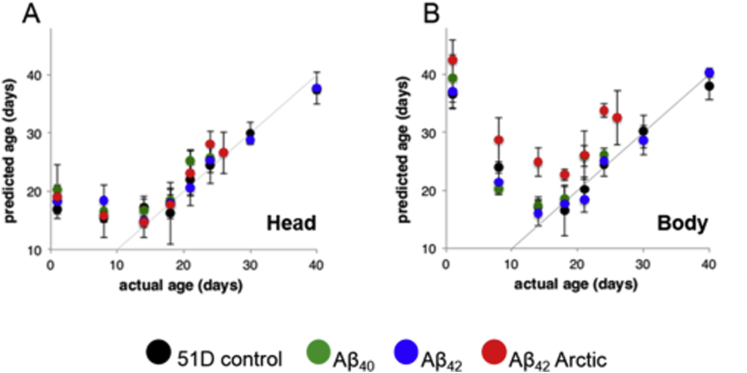


We also created an OPLS model, linking age to metabolic profiles, based on data from days 14 to 40 control and Aβ_42_ flies. This model was used to estimate the “metabolic age” of flies expressing Aβ_40_ and Aβ_42_ Arctic, which were not included in the model. The predicted ages for these flies corresponded closely to calendar age except for systemic samples from Aβ_42_ Arctic flies that consistently appeared to be 5 days older than expected (Supplementary Fig. 5A).

### 3.7 Metabolic changes are associated with Aβ expression and Aβ toxicity

**Fig. 8:**
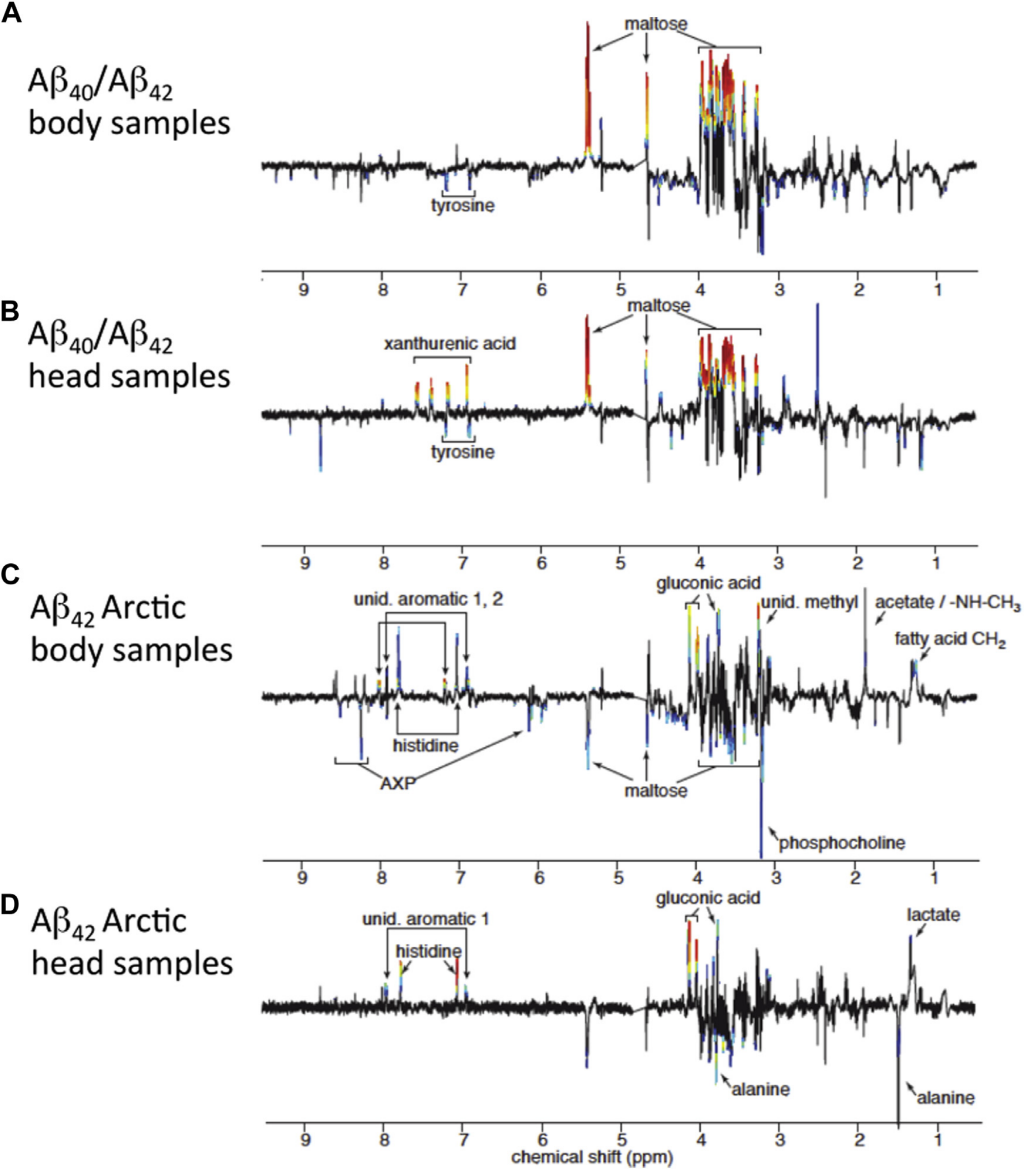
Summary of NMR spectral changes induced by the expression of A40/Aβ42 and Aβ42 Arctic. The figure provides an overview of the metabolomic changes induced by Aβ-expression. Positive and negative signals represent the increases and decreases in metabolite concentrations. Significant alterations were color coded from blue to red, with red representing the highest correlation between metabolite and genotype. Panels A and B compare the spectral changes between the 51D control and Aβ40/Aβ42 samples (comparing controls with nontoxic A expression), whereas panels C and D compare the changes between Aβ40/Aβ42 and Aβ42 Arctic flies (comparing non-toxic with toxic-A expression). Abbreviations: Aβ, amyloid beta; NMR, nuclear magnetic resonance.

**Fig. 9:**
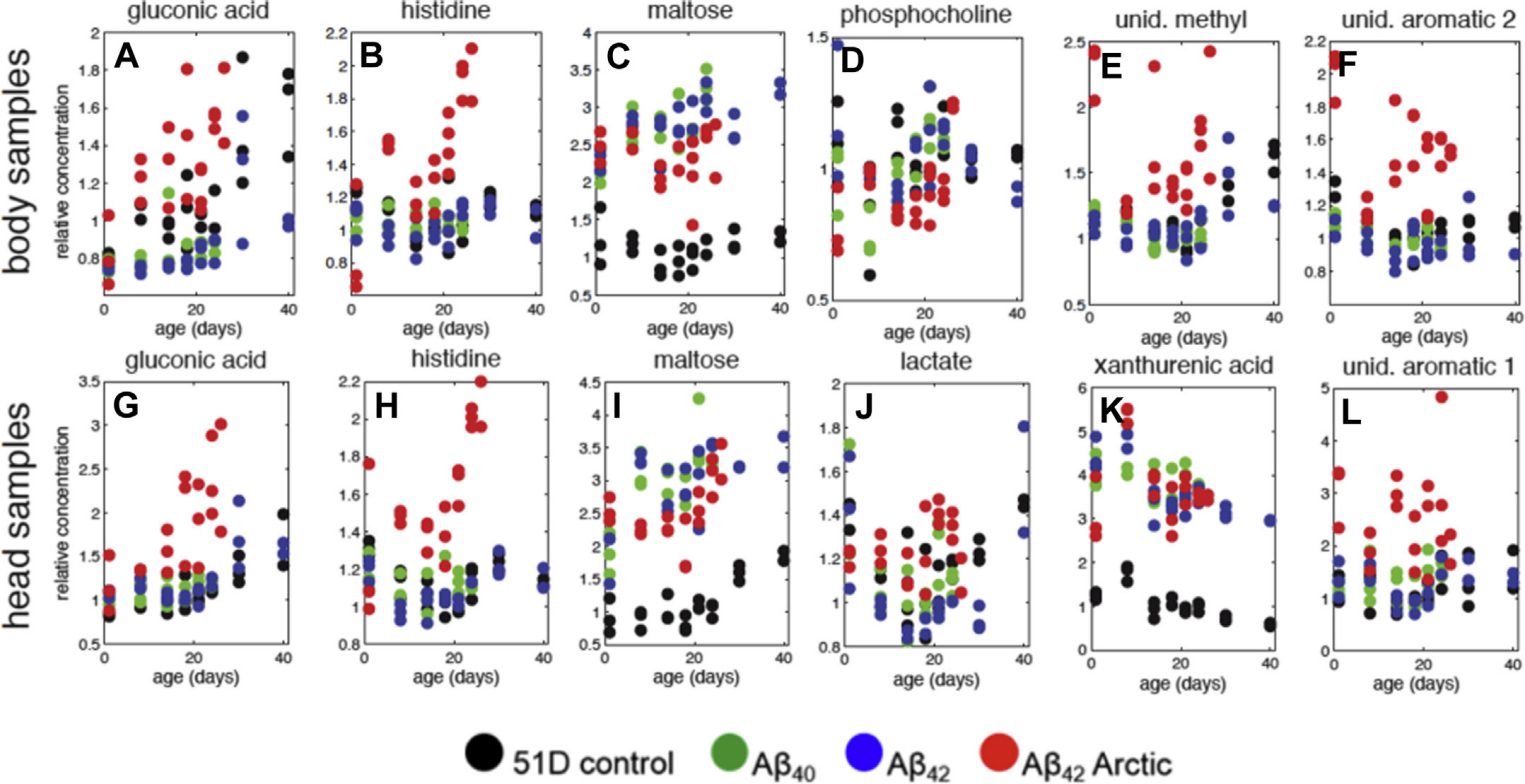
Relative metabolite changes in *Drosophila* head and body samples with age. The panels present fold changes in concentration relative to 1-day-old 51D control flies for selected metabolites from body (panels A–F) and head (panels G–L) samples. Concentrations for gluconic acid (A, G), histidine (B, H), and maltose (C, I) are shown for both body and head samples. Phosphocholine (D), an unidentified methyl group (E) and unidentified aromatic compound 2 (F) are shown for body samples, whereas lactate (J), xanthurenic acid (K), and unidentified aromatic compound 1 (L) are shown for the head samples. The individual panels display values for all replicate samples for each age and genotype. Note that for metabolites that have low concentrations in 1-day-old 51D control flies (A, E–G, K–L), the relative increase can be underestimated because of contributions from overlapping stronger signals.

Next, we identified which key metabolites accounted for the metabolomic signals that are apparent in Fig. 7. First, we compared controls with Aβ_40_ and Aβ_42_ flies to determine the Aβ-linked metabolic changes in the absence of a significant longevity phenotype. These flies have successfully adapted to protein aggregation stress and so metabolic changes may represent adaptive cellular responses. In the presence of Aβ_40_/Aβ_42_, there was a fourfold increase in maltose (Fig. 8 panels A and B, chemical shifts 5.40, 4.64 ppm *inter alia*, Fig. 9 panels C and I) and a decrease in tyrosine (7.18, 6.88 ppm, Fig. 8 panels A and B) in both head and body samples. In the head extracts, there was a marked increase in xanthurenic acid (7.53, 7.37, 7.16, and 6.92 ppm Fig. 8B, Fig. 9K) on Aβ_40_/Aβ_42_ expression.

Second, we compared Aβ_40_ and Aβ_42_ with Arctic Aβ_42_ flies to identify how the metabolome specifically reported decompensated protein aggregation. Flies expressing the toxic Arctic Aβ_42_ isoform showed a distinct series of metabolic changes when compared with those expressing Aβ_40_/Aβ_42_. In particular, the levels of gluconic acid (4.10, 4.01, 3.75 ppm Fig. 8 panels C and D, Fig. 9 panels A and G) and histidine (7.77, 7.05 ppm Fig. 8 panels C and D, Fig. 9 panels B and H) were increased in older (14–24 days) Arctic Aβ_42_ expressing flies. Although maltose levels were still much higher than in control flies, they were lower than in Aβ_40_ and Aβ_42_ expressing *Drosophila* (Fig. 8, panels C and D, Fig. 9 panels C and I). Specific changes in fly heads were higher lactate (1.31 ppm Fig. 8D, Fig. 9J) and lower alanine (3.77, 1.47 ppm Fig 8D). In fly bodies acetate (1.90 ppm) and fatty acid CH_2_ groups (1.26 ppm) were found to be increased (Fig. 8C), whereas adenosine nucleotides (AXP; 8.52, 8.26, 6.13 ppm) and phosphocholine levels (4.17, 3.58, 3.21 ppm) were reduced (Figs. 8C and 9D). In both heads and bodies signals from unidentified aromatic compounds (7.93, 6.93 ppm and 8.03, 7.80, 7.20, 6.90 ppm for heads and bodies, respectively) appeared in NMR spectra from the Arctic Aβ_42_ expressing flies (Fig. 9 panels F and L). These signals could not be unambiguously assigned, but when the chemical shifts were matched against the human metabolome database (Wishart et al., 2013), 8 of the 10 best matches were hydroxylated, carboxylated, or chlorinated compounds. As an example, the ^1^H next to a hydroxyl substituted carbon typically appears around 6.9 ppm. An additional signal, which likely corresponds to a methyl or methylene attached to an aromatic ring, appeared at 3.25 ppm.

The concentrations of a number of key metabolites were age dependent and/or Aβ dependent. The compounds that most faithfully reported the presence of Aβ, irrespective of lethality, include maltose, in the head and body and xanthurenic acid levels in the fly head (Fig. 9 panels C, I, and K) spectra. Both of these metabolites were increased in flies expressing Aβ_40_, Aβ_42_ and Arctic Aβ_42_, as compared with controls. Metabolomic changes specifically induced by the expression of the toxic Arctic Aβ_42_ included age-related increases in gluconic acid, histidine, and the appearance of unidentified aromatic compounds in both head and body samples (Fig. 9 panels A, B, E, F, G, H, and L). Levels of lactate were also higher in head samples (Fig. 9 panel J), and levels of AXP and phosphocholine lower in body samples (Fig. 9 panel D), in Arctic Aβ_42_ as compared with Aβ_40_/Aβ_42_ flies.

## 4 Discussion

AD is one of the most pressing age-related disorders facing society. Aging may be considered as the time-dependent accumulation of deficits in protective functions that result in a multisystem syndrome defined by increased fragility and propensity to die (Kirkwood and Austad, 2000, Partridge and Gems, 2002). In many organisms, this produces a log-linear increase in mortality rates with time (Curtsinger et al., 1992, Partridge et al., 2005). In some respects, the gradient of the mortality trajectory may be understood as the rate of physiological aging with chronological time. *Drosophila* aging can be slowed by reducing the ambient temperature and delayed by restricting calorie intake (Mair et al., 2003, Partridge et al., 2005). One important contributor to increased age-related fragility is a progressive degradation in the structural integrity of proteins. Such deficits in proteostatic mechanisms have been described primarily in *C. elegans*, but a similar process is thought to underpin the strongly age-related incidence of protein misfolding disorders such as Alzheimer's, Parkinson's and other, systemic, amyloid diseases (Chiti and Dobson, 2006, Taylor and Dillin, 2011).

In this study, we have used our Aβ-expressing *Drosophila* as a model of AD to probe how protein aggregation may modulate mortality. We asked the question whether a generalized increase in protein aggregation propensity is a cause or a consequence of the aging process. Our initial experiments indicated that expression of the lethal Arctic Aβ_42_ increased the gradient of the mortality trajectory. To our surprise, we observed that extrapolation of the plots to early life indicated that Arctic flies diverged from the control flies at a point around day 15 of adult life (Fig. 1B). This implies that young *Drosophila* before this time point are robust with respect to protein misfolding stress and that they becomes susceptible to the lethal effects of protein aggregation during midadult life. We tested this hypothesis directly using an inducible version of the fly model in which Aβ expression only occurred on oral dosing with RU486. In the first set of experiments (Fig. 2A), when Aβ was expressed in 15 days intervals, we found that the earliest, days 1–15, exposure window had only minimal consequences for the flies; exposure to Aβ later in life resulted in a reduction in the median survival. In the second set of experiments (Fig. 2B), we compared 4 cohorts of flies: those in which Aβ was expressed early (days 1–15), late (after day 15), continuously, or never. In this study, the lifespan of flies expressing Aβ only after day 15 was essentially identical to those where Aβ expression was continuous. This in turn indicates that there was little or no contribution of early Aβ to the accumulated deficits later in life. To quantify the early and late contributions of Aβ expression to mortality, we plotted and compared the 4 mortality trajectories (Fig. 2C). Remarkably, the gradient of the mortality trajectories was determined by whether Aβ was induced after day 15, whereas early Aβ expression resulted in a displacement along the y-axis. Assuming that this displacement accumulated steadily over the first 15 days of life, we can estimate that older flies were 50% more susceptible to the lethal effects of the peptide, as compared with the young population.

The differential effects of early and late Aβ exposure might be because of the way in which the organism handles the peptide at different stages of its life. To investigate this possibility, we collected flies from the 4 cohorts at various ages and made both total- and SDS-soluble Aβ extracts. We observed that early expression of Aβ led to the accumulation of soluble material, but this was quickly cleared, provided that expression was stopped at day 15 (Fig. 3A and Supplementary Fig. 7A); otherwise, continued expression resulted in the conversion of this soluble peptide into insoluble isoforms (Fig. 3B and Supplementary Fig. 7B). It is remarkable that soluble Aβ is lower in flies following induction at day 15 as compared with continuous expression. That these 2 groups of flies have identical longevity suggests that toxicity is generated by after day 15 insoluble peptide that is elevated similarly in both. This observation stands in contrast to the current consensus in the literature where small, SDS-soluble, oligomers (dimers) are thought to be the primary toxic Aβ species (Shankar et al., 2008). Overall, our findings support the study by Rogers et al (Rogers et al., 2012) who observed acute Aβ expression in older (>20 days) flies results in higher levels of Aβ and stronger reductions in longevity as compared with Aβ expression in younger flies.

**Supplementary Fig. 6:**
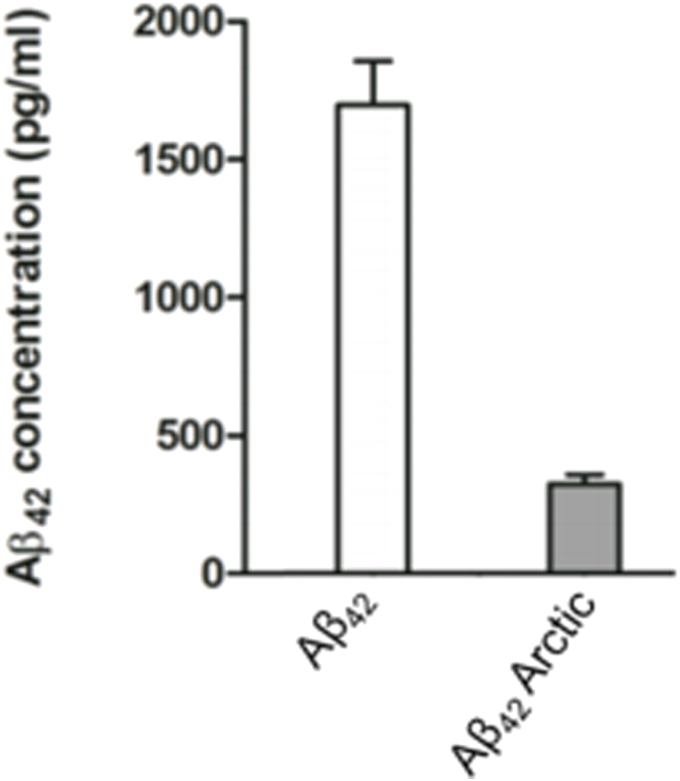


**Supplementary Fig. 7:**
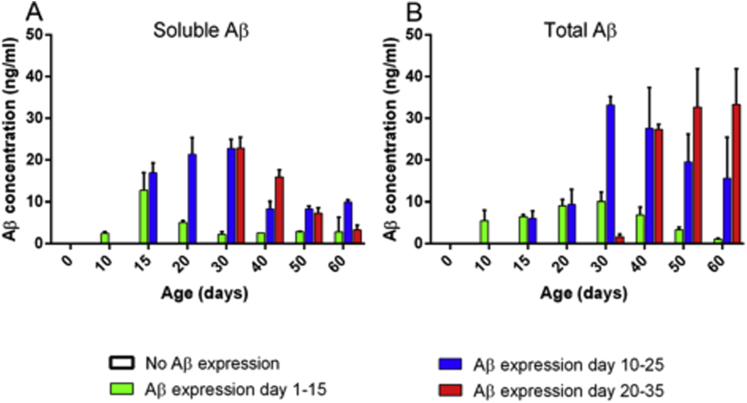


These data give us a picture of an organism that is robust in youth, but having passed an age threshold, becomes sensitive to Aβ toxicity; thereafter, the peptide seems to accelerate the aging process. Before this threshold age, the peptide appears to be held in a less-toxic, soluble, and labile conformation, and its subsequent conversion into insoluble material is associated with increasing mortality. There is evidence, particularly in *C. elegans*, that the stress placed on cells by such protein misfolding and deposition may unmask temperature-sensitive phenotypes. Specifically age and poly-glutamine expression sensitise the worm to the paralysis caused by a temperature-sensitive dynamin mutation (Ben-Zvi et al., 2009, Gidalevitz et al., 2006). Similar age-related protein destabilization is also seen in *Drosophila*, and so we investigated its role in the increased Aβ-related sensitivity seen in older flies (Demontis et al., 2013). Using a novel temperature titration assay in *shibire*^*TS2*^ flies that, like the worm carry a temperature-sensitive dynamin mutation (Chen et al., 1992, van der Bliek and Meyerowitz, 1991), we found that locomotion slowed progressively with increasing ambient temperature (Fig. 4). As predicted by the proteostatic hypothesis, we found that older *shibire* flies became paralyzed at progressively lower temperatures. However, under conditions where Arctic Aβ_42_ accumulated in brain sections, with lethal consequences (Supplementary Figs. 4 and 6); there was no effect on the paralysis titration profiles (compare Fig. 4, panels A with C). Assuming that the increased sensitivity of older *shibire* flies to heat-induced paralysis is reporting proteostatic decompensation, we conclude that Aβ accumulation and deposition is not an effective proteome-destabilizing factor.

Although Aβ appears not to exert its aging effects by accelerating a proteostatic collapse, our previous work has indicated that metabolic consequences, not least oxidative stress, may be responsible (Rival et al., 2009). To test whether we could detect an early metabolic effect of Aβ expression, we challenged flies with hydrogen peroxide in their food. In particular, we tested how flies, expressing Aβ_40_ or Aβ_42_, responded to oxidative stress before and after the day 15 threshold. We observed an age-related increase in hydrogen peroxide sensitivity in control and Aβ_40_ expressing flies. Remarkably, we also found that day 2 flies, expressing the Aβ_42_ isoform, were already as sensitive to oxidative stress as day 20 flies. These data are compatible with there being a biochemical signal, rather than a proteostatic defect, underpinning the transition from the robustness of youth to the fragility of older age. Surprisingly, we also noticed that 20-day-old control flies were more sensitive to the peroxide challenge than Aβ_40_ expressing flies. Although the precise mechanisms for this need further investigation, we speculate that the presence of nontoxic Aβ_40_ can act as a mild stressor and lead to an increase in reactive oxygen species defence without triggering Aβ toxicity.

To systematically investigate the metabolic changes associated with Aβ expression, we made extracts of water-soluble biochemicals from fly tissues at various ages and subjected them to analysis by NMR spectroscopy. The flies were separated into heads, to allow us to measure the local consequences of Aβ production, and bodies, where systemic metabolic changes could be assessed. Initially, the NMR spectra were subjected to PCA to provide an unbiased summary of the Aβ- and age-dependent metabolic changes (Fig. 6). We first noted an age-related trajectory that was largely similar for all genotypes. The metabolome of all *Drosophila* variants changed most rapidly during the first 8 days of life, whereas there was a smaller rate of change in the older flies. However, the expression of any Aβ isoform, whether it is Aβ_40_, Aβ_42_, or Arctic Aβ_42_, resulted in a displacement in the age-related trajectories away from the control population. This displacement was already evident from the earliest time points, indicating that the expression of Aβ results in early metabolic alterations.

Our next OPLS-DA model helped us visualize the differences between 3 groups of flies: (1) healthy controls, (2) asymptomatic flies expressing the aggregation-prone Aβ_42_, and (3) flies expressing the lethal Arctic Aβ_42_ (Fig. 7). We observed that the systemic metabolome of the oldest control and Aβ_42_ expressing flies changed so that it became more similar to, albeit younger, Aβ_42_ Arctic flies, indicating a related systematic response between aging and Aβ toxicity. Likewise, Aβ_42_ Arctic flies appeared older than other genotypes in an OPLS model of age in the systemic metabolome (Supplementary Fig. 5).

We then identified a set of metabolites that could be used to differentiate control flies from those expressing any isoform of Aβ (Fig. 8 panels A and B), and a distinct set (Fig. 8 panels C and D) that could differentiate flies expressing Arctic Aβ_42_, from flies with a normal life-expectancy (controls, Aβ_40_ and Aβ_42_). These 2 orthogonal sets of metabolites provided a 2-way differentiation in a nervous environment from heads and for the systemic metabolites from the bodies.

Remarkably, all Aβ-expressing flies exhibited a shift in carbohydrate metabolism, with maltose, a dietary glucose disaccharide, increasing 3-to 4-fold in both head and body extracts (Fig. 9 panels C and I). Similar increases were observed in *Drosophila* in response to both acute stress and artificial selection for stress resistance (Malmendal et al., 2013, Overgaard et al., 2007); interestingly, maltose has been suggested to have a chaperone-like function for proteins and membranes (Kaplan and Guy, 2004, Pereira and Hunenberger, 2006). Levels of xanthurenic acid were raised in flies expressing all 3 Aβ isoforms (Fig. 9K) indicating potential involvement of the kynurenine pathway that generates vitamin B3 from tryptophan, using vitamin B6 as a cofactor. Low levels of vitamin B6 result in raised proximal intermediates that are converted, by oxidation, to xanthurenic acid. It is notable that such intermediates as kynurenine and 3-OH-kynurenine are raised, as a ratio to tryptophan, in patients with AD (Gulaj et al., 2010, Schwarz et al., 2013). Furthermore, several studies have also shown that vitamin B therapy is protective in AD (Douaud et al., 2013).

The metabolites that separate Arctic Aβ_42_ flies from the other isoforms can be divided into those that are deviating from day one and those that mainly change after day 14 and deviate further with age. The unidentified, potentially hydroxylated, carboxylated, or chlorinated aromatic compounds appeared in both heads and bodies of Arctic Aβ_42_ flies, already from day 1 (Fig. 9 panels E, F, and L). If these are end products of oxidative stress it provides an interesting perspective on the H_2_O_2_ challenge experiments, since it indicates that the Aβ-induced chemical stress is taking place already from day 1 and with rather constant intensity. Decreased AXP levels in the bodies (Fig. 8C) follow a similar pattern and indicate that there is a reduction in adenosine triphosphate production, and a resulting loss in total adenosine phosphate, through deamidation of adenosine monophosphate to inosine monophosphate, already from day 1.

In contrast, gluconic acid was increased from around day 8 in bodies and day 14 in heads (Fig. 9 panels A and G). Accumulation of this metabolite was induced by severe oxidative stress and apoptosis in breast cancer cells (Morvan, 2013), and a 100 fold increase was induced by oxidative stress in *Arabidopsis* (Chaouch et al., 2012, Han et al., 2013). An increase in gluconic acid may either reflect oxidation of glucose or enhanced production of 6-phosphogluconate through the oxidative pentose phosphate pathway. When yeast is submitted to oxidative stress, part of the central carbohydrate metabolism is rerouted away from respiratory pathways into the oxidative pentose phosphate pathway to provide reducing equivalents to support antioxidant metabolism (Godon et al., 1998). The results are consistent with the view that the tricarboxylic acid cycle is impacted significantly by Aβ, resulting in oxidative stress (Baxter et al., 2007, Noctor et al., 2015).

Histidine is increased from day 1 in heads and from day 8 in bodies, and the increase is accelerated between days 14 and 26 (Fig. 9 panels B and H). This amino acid is a precursor for histamine and carnosine biosynthesis and a powerful antioxidant and anti-inflammatory factor (Peterson et al., 1998). Low levels of histidine have been associated with inflammation and oxidative stress in humans (Niu et al., 2012, Watanabe et al., 2008). Moreover, histidine is increased in urine from patients with Parkinson's disease (Luan et al., 2015).

Aβ-expressing flies had lower systemic levels of phosphocholine (Fig. 9D), reflecting similar reductions, in most brain regions, in a murine model of AD (Salek et al., 2010). By contrast, phosphocholine levels were higher in blood from patients with the earliest stages of AD (Mapstone et al., 2014).

Although still much higher than in control flies, maltose decreases to levels below Aβ_40_ and Aβ_42_ in middle-aged Aβ_42_ Arctic flies (Fig. 9 panels C and I). Abnormalities in sugar metabolism combined with raised lactate levels in fly heads are indicative of defects in aerobic respiration, and this is further supported by the reductions in AXP levels in the body extracts. Similarly, lactate is increased in brains of mice that model AD (Salek et al., 2010).

The changes in histidine (Fig. 9 panels B and H) and gluconic acid (Fig. 9 panels A and G) levels are remarkable, because they occur just at the time when the flies were undergoing the transition from robust youth to fragile older age. In this respect, they are potential biomarkers of the metabolic fragility that accompanies the lethal effects of Aβ accumulation. The aromatic oxidation products (Fig. 9 panels E, F, and L) and the changes in AXP, on the other hand, appear already from day 1 in agreement with the increase susceptibility to H_2_O_2_ challenge already at an early age.

**Fig. 10:**
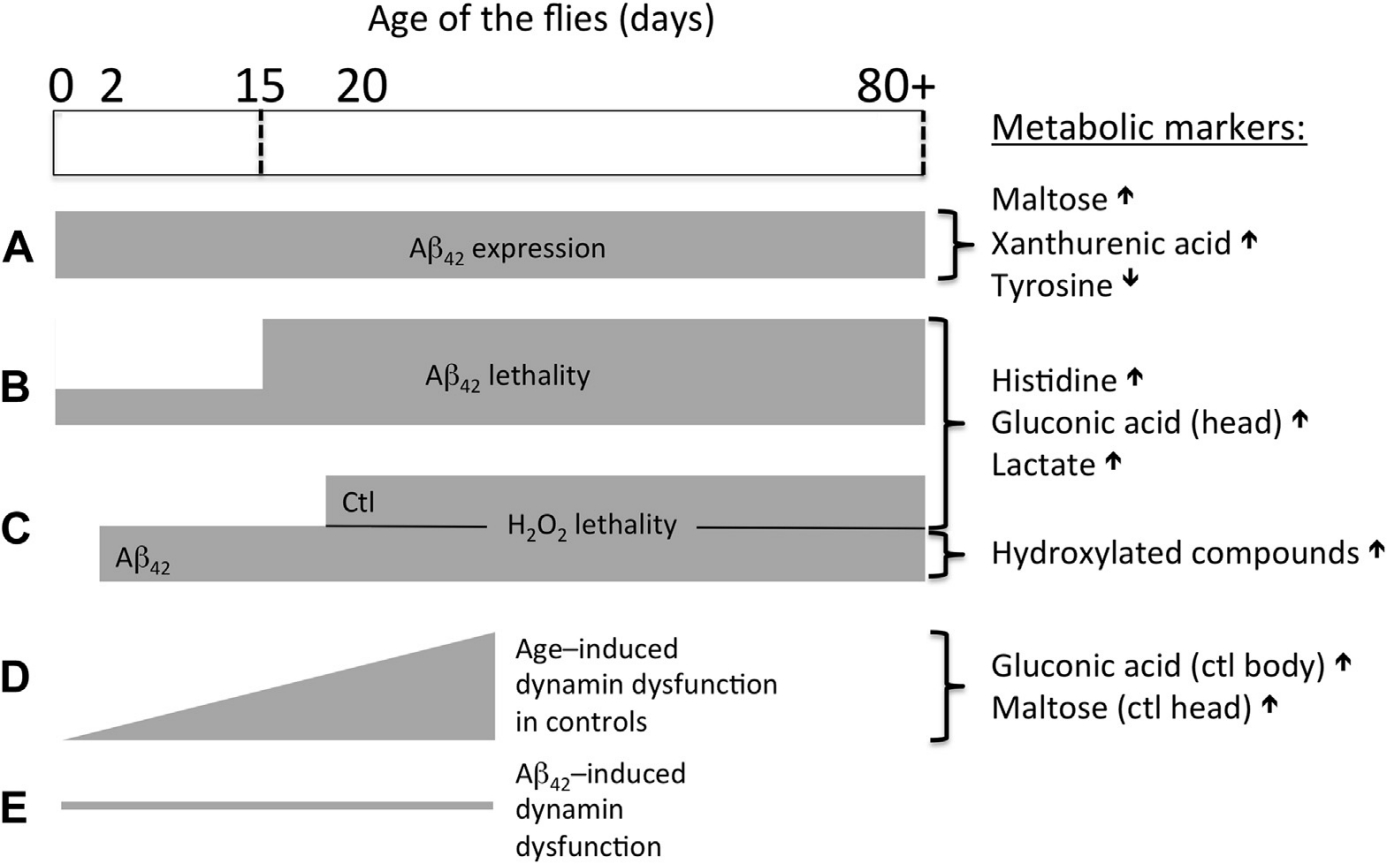
A timecourse of A expression alongside metabolomic correlates of experimental phentoypes. The timecourse of Aβ expression (A) is plotted alongside consequent experimental phenotypes (B–E). The increasing sensitivity of the flies to Aβ toxicity at day 15 (B) was closely correlated in time with sensitivity to an oxidative stressor in control flies (C, control). The presence of Aβ brings forward the onset of oxidative sensitivity (C, Aβ42). Thermal destabilization of dynamin, as a marker of proteostasis, occurs gradually with age across this timecourse (D). Aβ expression did not further functionally destabilize dynamin (E). Metabolic markers for A–D are presented (right column, ↑ = increase, ↓ = decrease). Metabolites that did not change with time (E) were not considered informative. Abbreviation: Aβ, amyloid beta.

The metabolite changes observed here (Fig. 10) only show a limited overlap with those detected in mouse (Salek et al., 2010) and man (Trushina and Mielke, 2013). There are multiple explanations: we use whole body parts rather than organs or body fluids, we separate effects of Aβ expression and Aβ toxicity, and we do of course use flies, and as it seems, the dominating effects here are those of decreased oxidative phosphorylation and increased oxidative stress.

In conclusion, whereas Aβ aggregation accelerates mortality in older flies, it does not seem to do this by exacerbating proteostatic collapse. Rather, the flies exhibit earlier oxidative stress, in response to Aβ expression that closely mirrors increases in mortality. The effects of this stress become obvious at older ages, inducing changes both in the lethality and the metabolomic profile.

## Disclosure statement

The authors have no actual or potential conflicts of interest.
