# Supplementary material for: Metabolic changes may precede proteostatic dysfunction in a Drosophila model of amyloid beta peptide toxicity
Source: Neurobiol Aging. 2016 May;41:39–52. doi: 10.1016/j.neurobiolaging.2016.01.009 (PMC4869574; doi:10.1016/j.neurobiolaging.2016.01.009)
Supplement: Legend for Supplementary Figs. 1–7 and Supplementary Table 1 [file mmc1.docx]

**Supplementary figure captions**

**Figure S1**: Expression of aggregation-prone Aβ_42_ Arctic leads to the formation of amyloid deposits

The panels show confocal images of fly brains expressing Aβ pan-neuronally from transgenes inserted at the 51D genomic insertion site. Aβ aggregates are visualized in red (6E10 anti-Aβ antibody) and the background staining (DNA staining with TOTO-3) in blue. All images represent *z*-stack projections that were taken with the same microscope settings with 10 x magnification. *Drosophila* were crossed and aged to 15 days at 25°C prior to brain dissection.

**Figure S2**: Aβ expression levels do not decline significantly with age

Quantitative PCR for Aβ mRNA was performed on samples from *Drosophila* of different ages. RNA was extracted from 80 flies for each genotype. Expression levels of the house-keeping gene *RP-49* were used as the background control. A two-way ANOVA did not reveal significant differences in gene expression levels with time. Error bars show the standard deviation of at least four replicate samples.

**Figure S3**: The calibration of RU486 levels for temporal Aβ expression

The panels illustrate the changes in total Aβ_42_ levels throughout ageing, as measured by MSD ELISA. A two-way ANOVA followed by a Bonferroni Multiple Comparison Test was carried out to test whether the two different induction time points resulted in altered Aβ concentrations at each particular age. Significant differences in Aβ concentrations were observed in panels A and B, where the expression of Aβ was induced by feeding the flies 12.5 µg and 50 µg RU486 respectively. No differences were detected when Aβ induction was achieved by feeding the flies 200 µg RU486 (panel C) and this concentration was subsequently chosen for further experiments (ns: not significant; **p* < 0.05; ****p* < 0.001; error bars show the standard error of mean of six replicate samples). The flies were maintained at 25°C throughout the experiment.

**Figure S4**: Aβ_42_ Arctic expressing flies that were maintained at 18°C show a decrease in longevity

Flies expressing Aβ_42_ Arctic pan-neuronally died significantly earlier (median survival = 108 days) than Aβ_40_ expressing *Drosophila* (median survival = 124 days) or 51D controls (median survival =131 days). A comparison of survival curves by the Log-Rank test revealed that the survival distributions between the control and Arctic Aβ_42_ expressing flies were significantly different (*p* < 0.001), while no such differences were detected between the control and Aβ_40_ expressing organisms (*p* = 0.35). All *Drosophila* populations were crossed and maintained at 18°C throughout life. The experiment was carried out with at least 90 flies per genotype.

**Figure S5**: OPLS predicts an increased rate of metabolic ageing in Aβ_42_ Arctic body samples

The panels illustrate age predictions made by an OPLS model based on the data from 14-40 day old 51D control and Aβ_42_ samples. Values for samples included in the model were estimated by cross validation. Predicted age equals actual age along the grey line. The data points represent the mean value of tree replicate samples and the error bars show the standard deviation

**Figure S6**: Aβ concentrations in 35 days old *Drosophila shibire^TS2^*

The figure shows the amount of total Aβ in *shibire^TS2^* flies that were used in climbing experiments. The flies were crossed and maintained at 18°C. The Aβ measurement was performed with 3 biological replicate samples for each genotype. Error bars show the standard deviation.

**Figure S7**: Aβ accumulation in fly heads at different time points

The levels of SDS-soluble and total Aβ in fly head homogenates were measured by MSD ELISA throughout ageing (see figure 3 for additional information). Error bars show the standard deviation of at least three biological replicates.

**Supplementary tables**

**Table S1A:**

The vertical displacement of the each of the paired lines in fig. 2C (open vs. closed rectangles and open vs. closed circles) provides two estimates of the accumulated mortality trajectory displacement due to Aβ expressed between days 1-15. Assuming that displacement of the mortality trajectories accumulated linearly over 15 days the daily displacement during days 1-15 can be calculated.

| Table S1A: Aβ-attributable gradient increase for the hypothetical day 0-15 mortality curves | | | | |
| --- | --- | --- | --- | --- |
| Aβ expression day 15 onwards | **+** | **+** | **-** | **-** |
| Aβ expression day 0-15 | **-** | **+** | **-** | **+** |
| Average displacement of mortality trajectory | 1.17 | | 0.69 | |
| Aβ-attributable gradient increase for days 0-15 | **0.08 day^-1^** | | **0.05 day^-1^** | |

**Table S1B:**

The difference in the gradients between each of the paired lines in fig. 2C (filled rectangles vs. filled circles and empty rectangles vs. empty circles) provides two estimates of the daily excess displacement of the mortality trajectories due to Aβ expression from day 15 onwards.

| Table S1B: Aβ-attributable gradient increase for the day 15+ mortality curves | | |
| --- | --- | --- |
| Gradient of adult mortality trajectory | Aβ day 0-15 | No Aβ day 0-15 |
| No Aβ day 15+ | 0.12 day^-1^ | 0.14 day^-1^ |
| Aβ day 15+ | 0.22 day^-1^ | 0.23 day^-1^ |
| Aβ-attributable gradient increase for days 15+ | **0.10 day^-1^** | **0.09 day^-1^** |
